# Neuroinflammation and nutrition in Alzheimer’s disease

**DOI:** 10.3389/fneur.2025.1622571

**Published:** 2025-07-23

**Authors:** Hyoungshin Park, Mya Ni, Yvonne Le

**Affiliations:** Department of Biology, School of Arts and Sciences, Massachusetts College of Pharmacy and Health Sciences, Boston, MA, United States

**Keywords:** neuroinflammation, Alzheimer’s disease, microglia, diet, short chain fatty acids, neurodegeneration

## Abstract

The brain contains approximately 100 billion neurons and over 200 billion glial cells, which are integral to the neuronal networks that support normal brain function in the central nervous system. The complexity of the brain makes the diagnosis and treatment of neurodegenerative disease particularly challenging. Neuroinflammation and neuronal cell death contribute to the development of neurodegenerative diseases such as dementia. Dementia refers to a decline in memory and thinking ability, affecting approximately 55 million people worldwide. Owing to the association of multiple factors, including amyloid-*β* plaque, tau-fibrillary tangles, neuroinflammation, nutritional defects, and genetic mutations, the exact cause of the most common type of dementia, Alzheimer’s disease, remains elusive. These multiple factors may cause damage to neurons and glial cells, leading to neurodegeneration. Very few therapeutics are available for neurodegenerative diseases due to the limited understanding of their pathogenesis, resulting in the lack of biomarkers and drug targets. Recent attention has shifted toward addressing modifiable risk factors such as unhealthy diets and lifestyles to delay the onset of Alzheimer’s disease. Unhealthy diets that consist of saturated fatty acids and refined sugars, with other multiple risk factors, increase neuroinflammation and oxidative stress, furthering cognitive decline and progression of neurodegeneration. Mitigating these risk factors with antioxidants, anti-inflammatory-based nutrition, and multidomain lifestyle intervention, which may include physical exercise, cognitive stimulation, and social engagement, may delay the development of neurodegenerative diseases and cognitive decline. In this review, we focus on the role of neuroinflammation in contributing to neurodegeneration and dietary influence in Alzheimer’s disease.

## Introduction

Neurons and glial cells construct neural networks and synapses, maintaining a healthy microenvironment within the brain. Glial cells provide essential support for the axonal function and synaptic plasticity of neurons, and they participate as integral mediators of neuronal networks in the central nervous system (CNS) (1, 2). Specifically, astrocytes and microglia are the primary supporting glial cells in the CNS, where they respond to stress, infection, and injury, and survey the microenvironment (3).

Neurodegeneration is the progressive loss of functional neurons, often triggered by neuroinflammation, oxidative stress, glial activation, and cerebrovascular damage. It ultimately results in neuronal cell death and is associated with neurodegenerative diseases such as Alzheimer’s disease (AD). The pathological features of these diseases include inflammation, genetic defects, altered energy metabolism, abnormalities in cytoskeletal structure and proteostasis, synaptic network defects, and pathological protein aggregation (4). Neuroinflammation is triggered by various insults, including infection, toxic metabolites, stress, or metabolic disturbances, which activate microglia to a proinflammatory stage, resulting in the secretion of proinflammatory cytokines. Without clinical interventions, neuroinflammation can lead to neurodegeneration, disability, and, ultimately, death. Although the causes and pathological mechanisms of AD are not yet clearly identified, many associated causes and noticeable pathologies of AD have been established. The three major pathologies in AD patients are (i) accumulation of amyloid-*β* (Aβ) particles in the brain, (ii) tubulin associated unit (tau) fibrillation in the neurons, and (iii) hyperactivated microglia and neuroinflammation in the brain (5, 6). The risk factors of AD include non-modifiable and modifiable factors, and the synergistic effects of these risk factors contribute to the development of AD (7). It is particularly crucial to address modifiable risk factors such as unhealthy diets and lifestyles to delay the development of AD.

The current stage of AD research and the remaining questions are briefly reviewed, followed by an examination of neuroinflammation and the interactions between microglial activation and neuronal cell health in AD. Moreover, we examine well-established diets to delay and/or reduce the risk of AD. Finally, we review emerging gut microbiome research and the modulation of brain health through the regulation of communication between the gut and the brain. We aim to highlight the impact of healthy diets on AD and improved neuronal health.

## Alzheimer’s disease

AD affects over 7 million Americans, and its typical symptoms include memory loss, language problems, impaired reasoning, and aggressive behavior (8). Eventually, the shrinking of the brain cortex and atrophy will lead to cognitive failure (5). Single-nucleus RNA sequencing of human prefrontal cortex data suggests that AD pathology is associated with alterations in gene expression in synaptic signaling, chromatin organization, lipid metabolism, mRNA and tRNA metabolic processes, and mitochondrial function, suggesting multifaceted disease mechanisms (9). There are two types of AD: the first is familial AD, which is caused by dominant genes (e.g., the presenilin-1 and presenilin-2 genes and the amyloid precursor protein gene) and accounts for 5–10% of AD cases. The second type, accounting for 90–95% of AD, is sporadic AD, which is caused by a combination of genetic factors (e.g., polymorphisms of apolipoprotein E and variants of triggering receptors expressed on myeloid cells 2) and environmental factors, such as cardiovascular health, diet, physical activity, and social engagement (5, 10, 11). Although the cause of sporadic AD is poorly understood due to the complexity of the brain’s structure and function, age is the most significant risk factor for sporadic AD, considering that 50% of AD occurs in populations of 80 years and older. A combination of age, genetics, female sex, low education, neuroinflammation, cognitive inactivity, air pollution, unhealthy diet, and unhealthy lifestyle may lead to progressive AD (12).

Amyloid precursor protein (APP) is an integral membrane protein highly expressed in neuronal synapses (13). APP plays a role in nerve growth and repair after injury and is hypothesized to aid in nervous system development, synaptogenesis, and axonal growth guidance (14). In the normal brain, the turnover of APP occurs when *α*- and *γ*-secretases cleave APP into smaller peptides, specifically the 40-amino acid form A*β*40, which are soluble and subject to degradation. In the AD brain, however, β- and *γ*-secretases cleave APP into the 42 amino acid peptide Aβ42, an insoluble form of amyloid monomers, which are sticky and attract more Aβ monomers to form plaques near the neurons and interfere with synaptic signal transduction (5). Genetic mutations in APP proteins or mutations in the *γ*-secretase have been linked to AD (13). Targeting APP genes or γ-secretase for gene therapy might be an interesting therapeutic approach to reduce plaque accumulation.

Tau proteins have microtubule binding domains to stabilize microtubules and cytoskeleton structures, and regulate axonal transport and synaptic function in the neuron (15). In AD, tau filaments become hyperphosphorylated (i.e., p-tau), causing p-tau filaments to detach from microtubules due to a conformational change and contribute to neurodegeneration. The p-tau fibrils form clumps, recruiting more p-tau to create neurofilament tangles within the neuron. It is unknown why the cellular clearance mechanisms in neurons do not remove these abnormal proteins. The accumulation of these tau neurofilament tangles may interrupt electrical signaling in neurons, leading to neuron death. As tau molecules have been found in blood samples, the significance of tau modulation as a blood biomarker in AD has been emphasized for further investigation (16).

Resting microglia actively survey the cerebral environment to maintain brain homeostasis. Upon pathogen invasion or inflammation, microglia are activated and secrete cytokines to resolve inflammation. Activated microglia secrete either anti-inflammatory cytokines, such as interleukin-10 (IL-10) and IL-13, which contribute to tissue repair and neuroprotection (3), or pro-inflammatory cytokines, such as tumor necrosis factor-*α* (TNF-α) and IL-1β, which contribute to the pathogenesis and neurotoxicity. In AD, abundant hyperactivated proinflammatory microglia are localized around Aβ plaques and recruit reactive astrocytes, which have a detrimental impact on the microenvironment (17). The activated proinflammatory astrocytes are unable to remove Aβ plaques and further induce neurotoxicity (3). These observations suggest that modulating the proinflammatory reaction of microglia in the brain may be an effective treatment option for reducing AD risk and neuroinflammation.

## Neuroinflammation

Healthy glial cells secrete anti-inflammatory cytokines and play significant roles in neuroinflammation by providing defense against invading pathogens (18). Astrocytes are abundant glial cell types in the CNS, which help regulate fluid and ion homeostasis, controlling blood flow, protecting neurons from excitotoxic injury by clearing excessive neurotransmitters, promoting the formation of synapses, and contributing to the blood–brain barrier (BBB) construction and maintenance (19). Pathological attack and nerve injury activate astrocytes into an anti-inflammatory type through changes in their phenotype, function, and gene expression, which in turn lead to neuroinflammation (20). Proinflammatory cytokines reactivate astrocytes into a proinflammatory type, leading to the production of neurotoxic and neurodegenerative cytokines, as well as reactive oxygen species (ROS). Additionally, reactivated proinflammatory astrocytes upregulate the expression of the complement system, resulting in neurotoxicity. For example, reactive astrocytes secrete complement 3 (C3), which signals to the C3a receptor (C3aR) on neuronal membranes, thereby aggravating neuronal function (21). Targeted inhibition of the binding of C3 and C3aR on neuronal membranes might be a meaningful approach to mitigate neurodegeneration.

Astrocytes are the primary producers of Apolipoprotein E (ApoE), and the APOE genes encode three types of ApoE proteins (ApoE2, ApoE3, and ApoE4) that are involved in lipid metabolism as cholesterol transporters in the brain. APOE ɛ4 allele is a high-risk factor linked to impaired memory and cognitive decline, presenting astrocyte activation and higher accumulation of Aβ plaques in sporadic AD (3). ApoE4 protein may increase the sequestration of cholesterol and interfere with myelination in the brain, which is associated with AD pathology (22). Recently, the interaction between astrocytes and microglia has been recognized as a critical factor in both neuroinflammation and neurodegeneration. For example, the interaction of C3 from proinflammatory astrocytes and C3aR on proinflammatory microglia regulates microglial phagocytosis function (21). Therefore, it is imperative to understand the role of crosstalk between astrocytes and microglia in AD pathogenesis.

Microglia are the primary resident immune cells in the CNS. In the normal brain, microglia eliminate foreign molecules that cross the tightly guarded BBB membrane, which allows only small lipophilic molecules with low permeability to pass through and prevent the accumulation of cellular debris and unwanted proteins, such as Aβ amyloid (23). Microglia express the triggering receptor expressed on myeloid cell 2 (TREM2) on their membrane, where TREM2 modulates the phagocytosis of ApoE and Aβ amyloid plaques (11, 24, 25).

In hyperactivated microglia, however, proinflammatory cytokines are released, which, together with reactive proinflammatory astrocytes, may trigger destructive signals for neurons and lead to the accumulation of Aβ plaques and tau fibrillation in neurons (26). The hyperactivated microglia may exhibit reduced phagocytosis, resulting in the accumulation of Aβ42 amyloid plaques and problems with immunosurveillance in the brain, leading to neuroinflammation and neuronal death (3, 23, 27). Cell-based therapies targeting the depletion of activated microglia or the replenishment of healthy microglia may be an interesting therapeutic option (28). Another possible microglial function in the diseased brain may be the modulation of TREM2 receptor binding to Aβ plaques, which further recruits amyloid particles around neurons, ultimately leading to neurodegeneration in AD (24, 29). In addition, hyperactivated microglia may participate in the phagocytosis of insoluble p-tau and the spreading of p-tau through exosome secretion (11, 30). Although microglia interact with Aβ plaques via several mechanisms, including phagocytosis, immune hyperactivity, and exosome secretion, it is unclear whether microglial dysfunction is the cause or effect of AD (25, 29, 30).

Highly pathological neuroinflammation in the CNS is associated with activation of glia, production of proinflammatory cytokines and chemokines, infiltration of peripheral immune cells, edema, and increased BBB permeability and BBB failure (31). Multiple factors, including environmental toxins, toxic metabolites, infections, and stress, contribute to neuroinflammation. Air pollutants activate glial cells and induce oxidative stress and cerebrovascular damage in the CNS (32). Pesticides and heavy metals can cause cellular damage, triggering inflammatory responses that are associated with the production of ROS and neuroinflammation (33). Stressful life experiences are related to elevated proinflammatory cytokines, leading to the activation of cortical microglia and alterations in brain structure and function that increase the risk of neurodegenerative diseases (34). It remains unclear exactly how neuroinflammation drives the progression of neurodegenerative diseases, as it can have both protective and detrimental effects, depending on the context and stage of the disease.

## Dietary influences and neuroinflammation

Twelve modifiable risk factors account for more than 40% of worldwide dementia cases (35). These modifiable risk factors include less education, hearing loss, traumatic brain injury, hypertension, alcohol, obesity, smoking, depression, social isolation, physical inactivity, diabetes, and air pollution (8, 35, 36). Because these risk factors are interconnected, addressing several of them can lead to a significant improvement in cognitive function. Epidemiological studies suggest that dietary changes may protect against cognitive decline and dementia, and modifiable lifestyle and environmental factors, including cardiovascular health and physical activities, may influence AD development and pathology (11, 37, 38). Nutrition is an essential modifiable factor affecting cardiovascular health and metabolic risk that can delay cognitive decline and dementia in the aging population (39).

Three types of anti-inflammatory and antioxidant diets may be beneficial for brain health and lower AD risk: The Mediterranean diet (MeDi), dietary approaches to stop hypertension (DASH), and Mediterranean-DASH intervention for neurodegenerative delay (MIND) (40). MeDi is characterized as high in vegetable oils and low in saturated fat and emphasizes high intake of fruits, green leafy vegetables, whole grains, legumes, olive oils, nuts, and seeds, moderate consumption of fish, and low consumption of dairy products, alcohol, red and processed meats, resulting in neuroprotective effects assessed by cognitive tests such as episodic-, working-, semantic memory and visuospatial ability (41–43). High adherence to MeDi was associated with the preservation of brain structure and brain metabolic activity, as well as lower AD risk in American adults (44, 45). The DASH diet was developed to reduce blood pressure by consuming foods rich in potassium, calcium, and fiber, while limiting sodium, saturated fats, total fats, and cholesterol, and is associated with delayed cognitive decline and reduced AD risk (46). Hypertension is associated with reduced cerebral perfusion, leading to decreased oxygenation in the brain, causing brain atrophy and brain shrinkage, resulting in cognitive decline and increased risk for AD and dementia (47). DASH is based on a low intake of sodium to reduce the risk of hypertension and dementia and is characterized by a high consumption of fruits, vegetables, nuts, whole grains, and low-fat dairy products, as well as fish, and a low consumption of red and processed meats, tropical oils, sweetened beverages, and sweets (48). The MIND diet is designed to provide neuroprotection and reduce AD risk with slower cognitive decline. The MIND diet is a hybrid of the MeDi and DASH diets, incorporating additional brain-healthy food groups that have been purportedly linked to a decreased risk of dementia (49). The diet is based on the intake of 10 brain-healthy foods (i.e., leafy green vegetables, other vegetables, nuts, berries, beans, whole grains, fish, poultry, olive oil, and wine) to boost brain health and limit animal and high saturated fat, resulting in a significant reduction of AD risk (49). Additionally, the ketogenic diet (KD), a very high-fat and low-carbohydrate diet, can improve cognitive ability and quality of life in patients with mild to severe AD by using ketone as fuel for the brain (50–52). While dysfunctional glucose transporters in the brain may contribute to cognitive decline in AD, a ketone or a high-fat intake in the KD diet as an alternative energy source could reduce the oxidative burden for the brain (51, 52).

Neuroprotective bioactive compounds, such as omega-3 fatty acids, vitamin E, vitamin B, and choline, help maintain brain health and support optimal brain function. Immune modulators, including polyphenols, antioxidants, and unsaturated fats, may reduce the risk of systemic inflammation and oxidative stress and improve cognitive function and neuroinflammation in the brain (53–57). Docosahexaenoic acid found in fatty fish may reduce the accumulation of p-tau tangles and increase lipoprotein receptor 11, which in turn diminishes Aβ levels (54, 58). Polyunsaturated fatty acids found in vegetables, whole grains, nuts, seeds, and fruits may provide benefits against AD. Short-chain fatty acids (SCFAs, e.g., acetate, propionate, butyrate) are produced by the fermentation of dietary fibers by gut bacteria in the colon (59). SCFAs can enhance gut barrier integrity, regulate glucose and lipid metabolism, and modulate the immune system and inflammatory response (59, 60).

Additionally, modifiable lifestyles, including exercise, cognitive activity, social engagement, and systemic health determinants, can influence the development of AD (61). The Finnish Geriatric Intervention Study to Prevent Cognitive Impairment and Disability (FINGER) demonstrated that a multidomain lifestyle intervention can improve or maintain cognitive function in older adults at risk (38, 62, 63). An observational study of cognitive health in Black and White Americans and between genders found that participants with high adherence to MIND had a 4% reduced risk of cognitive impairment compared to the low adherence group (64). High adherence to healthy diets and healthy lifestyles may contribute to a reduction in cognitive decline and the risk of AD (36, 61).

## Gut microbiome and neuroinflammation

Bidirectional interaction between the gastrointestinal tract and the CNS allows signals to travel from the brain to reach the gut or from the gut to the brain through the gut-brain axis. The gut-brain axis may provide feedback for reducing neuroinflammation and oxidative stress (65). Additionally, the oral cavity serves as a primary gateway to the digestive tract and maintains a diverse population of microorganisms, the oral microbiota (66–68). The gut and oral microbiome play a significant role in determining how the diet can elicit effects on the whole body, including the CNS. A diverse microbiota produces metabolic byproducts, such as SCFAs, folate, and vitamin K, that influence various metabolic processes, neurotransmitter regulation, and immune signaling (60, 69).

The composition of microbiota in each individual is highly variable, and the diversity of microbiota in the gut is crucial for maintaining a healthy gut microbiome within the body (70, 71). The low microbial diversity or microbial imbalances in the gut are linked to neuroinflammation and AD (72–74). For example, 16S rRNA analysis of stool samples from AD patients revealed a lower gut microbiota diversity and a higher abundance of proinflammatory bacteria, such as *Escherichia* and *Enterobacter*, compared to controls (65, 72, 73). Gut microbiota regulates amyloid deposition, as evidenced by studies using antibiotic treatment in an amyloidosis mouse model and fecal microbiome transplant in a germ-free/gnotobiotic mouse model (75, 76). The antibiotic treatment in the amyloidosis mice leads to a lower Aβ amyloid plaques than the controls, and fecal microbiota transplantation methods into germ-free/gnotobiotic mice result in drastically a higher cerebral Aβ amyloid pathology than the controls (75, 76). In addition, treating the tauopathy mouse expressing human isoforms of APOE4 with antibiotics resulted in reduced tau pathology and decreased neurodegeneration (77, 78). Additionally, periodontitis is associated with low cognitive performance and AD, suggesting the oral microbiome may contribute to AD development (66, 67).

Dietary prebiotics are indigestible nutritional fibers that are fermented by gut bacteria in the large intestine, and produce SCFA. SCFA contributes to a decrease in neuroinflammation and a significant reduction in Aβ plaque deposition (79–81). The MeDi and DASH diets include abundant dietary fiber, which provides neuroprotective effects by increasing the diversity of microbiota and producing anti-inflammatory metabolites. The MeDi diet offers high fiber for the gut microbiota, which in turn produces SCFA metabolites that influence glial cells to adopt an anti-inflammatory function in the brain (46, 47). Moreover, the modified Mediterranean-ketogenic diet (MMKD), which allows an increased intake of vegetables and fruits, along with fats and proteins derived from healthy sources, alters the gut microbiota and increases beneficial SCFAs (50, 51).

Probiotics are live microorganisms present in certain fermented foods, such as yogurt, that play a pivotal role in restoring the gut microbiota’s composition, confer health benefits to the host, and influence host immune responses (82). Two common probiotics, such as *Bifidobacterium* and *Lactobacillus*, can help establish a healthy gut microbiome by strengthening intestinal barriers and reducing inflammation (83). For example, the traumatic brain injury mouse model, after receiving probiotic mixtures that included *Lactobacillus* species, reduced neuroinflammation and modified gut microbiome diversity (84). Modifying the gut microbiome composition and diversity through prebiotics and probiotics, therefore, may reduce neuroinflammation and delay AD.

## Discussion

AD is the most common type of dementia, accounting for over 120,000 deaths in the US in 2022 (85). The exact cause of the disease is still unclear. Still, many research efforts point to multiple risk factors such as genetics, age, neuroinflammation, and unhealthy diet and lifestyle contributing to AD development. Limited treatments are available to delay AD development, such as small molecules (e.g., glutamate modulators, acetylcholinesterase inhibitors) to improve synaptic function and immunotherapy (e.g., anti-A*β*: lecanemab, donanemab, or anti-β/*γ*-secretases) to remove plaques and reduce tau tangles, but have had modest success (7, 8, 86, 87).

The development of AD therapeutics is hindered by a lack of understanding of the causes of AD, the few diagnostic tools for early diagnosis, and difficulties in creating biomimetic clinical disease models (88). For example, the tau transgenic mice have significant limitations in accurately recapitulating the complexity of human tauopathy, such as age-dependent, anatomical changes seen in human AD (27). Emerging diagnostic technologies may help alleviate this situation, for example, blood tests based on Aβ and p-tau217 proteins have successfully predicted AD diagnosis with 88–92% accuracy (89). Alternative 3D human *in vitro* AD models that maintain spatial geometry and neuron-glial cell interactions seen in the human brain are being developed (90, 91). In addition, 3D human brain organoids provided the spatial architecture of the brain and enabled cell–cell interactions, recapitulating the neuron-glial cellular network (91). Identification of biomarkers and therapeutic targets for AD using biomimetic 3D human *in vitro*/organoid models may provide opportunities for better drug development (92).

Neuroinflammation acts as a double-edged sword in AD, providing beneficial anti-inflammatory and detrimental proinflammatory effects. In AD mouse models, depletion of microglia by inhibitors of colony-stimulating factor 1 receptor has been shown to reduce plaque accumulation, neuroinflammation, and improve cognition (93, 94). TREM2-activating antibodies can enhance microglial phagocytosis, leading to increased microglial activity around amyloid plaques and improved cognition in AD mice (95). Therefore, modulating neuroinflammation by timely regulation of microglial function using targeted anti-inflammatory drugs may be a practical approach to treating AD patients.

Addressing modifiable risk factors of AD may help improve cognitive health. A diet that enhances anti-inflammatory and antioxidant function, as well as cardiac health, is one of the most significant modifiable factors. MeDi, DASH, MIND, and MMKD diets may promote anti-inflammatory and antioxidant effects, neuroprotection, and contribute to healthy aging.

The oral and gut microbiome can trigger inflammation in the brain, increase BBB permeability, and amyloid plaque deposition, contributing to AD pathogenesis (96, 97). Maintaining a healthy oral and gut microbiome with prebiotics and probiotics will be crucial in delaying the onset of AD.
